# Low-Dose Radiotherapy for Patients with Pneumonia Due to COVID-19: A Single-Institution Prospective Study

**DOI:** 10.3390/biomedicines11030858

**Published:** 2023-03-11

**Authors:** Tomasz Wojciech Rutkowski, Jerzy Jaroszewicz, Damian Piotrowski, Krzysztof Ślosarek, Barbara Sobala-Szczygieł, Dorota Słonina, Bożena Włostowska, Dawid Bodusz, Maciej Piasecki, Michał Nachlik, Barbara Oczko-Grzesik, Adam Gądek, Dorota Kowal, Roman Rutkowski, Elżbieta Wojarska-Tręda, Krzysztof Składowski

**Affiliations:** 1 Maria Sklodowska-Curie National Research Institute of Oncology, Gliwice Branch, ul. Wybrzeże Armii Krajowej 15, 44-100 Gliwice, Poland; 2 Department of Infectious Diseases and Hepatology, Medical University of Silesia, 41-902 Bytom, Poland

**Keywords:** COVID-19, pneumonia, low dose radiotherapy, cytokine storm, inflammation

## Abstract

Purpose: Results of the low-dose radiation therapy (LDRT) in patients with pneumonia due to COVID-19 has been presented. Methods: Fifteen patients received a single-fraction radiation dose of 1 Gy to the bilateral lungs due to pre-ARDS pneumonia in the course of COVID-19. Follow-up was performed on days 1, 3, 5, 7, 14 after LDRT. Results: Eleven patients (73%) were released up until day 28. Median hospitalization was 20 days; 28-day mortality was 13%. Median O_2_ saturation improved within 24 h after LDRT in 14/15, with median SpO_2_ values of 84.5% vs. 87.5% *p* = 0.016, respectively. At day 14 of hospitalization, 46% did not require oxygen supplementation. Significant decline in CRP and IL-6 was observed within 24 h post LDRT. No organ toxicities were noted. Conclusion: LDRT is feasible, well tolerated and may translate to early clinical recovery in patients with severe pneumonia. Further studies are needed to determine optimal candidate, time and dose of LDRT for COVID-19 patients with pneumonia.

## 1. Introduction

In November, 2021, we searched PubMed using the search terms “COVID-19” and “low dose radiotherapy” [1]. The search found only seven studies presenting preliminary results of utilizing LDRT as a suppressor of COVID-19-related pneumonia. Although the most patients with SARS-CoV-19 infection are asymptomatic or present only mild symptoms, in about 10–20% of cases the infection may progress to interstitial pneumonia. Alveolar damage caused by inflammatory infiltrates subsequently leads to acute respiratory distress syndrome (ARDS) compromising gas exchange and forcing mechanical ventilation. At this phase of disease, high mortality rates are observed and even 60–80% of such patients will die [2,3,4]. Up to now, no effective, universal treatment against this proinflammatory cascade has been presented. Furthermore, currently recommended anti-cytokine therapies, particularly tocilizumab, show effect mainly in selected patients (ie., with serum IL-6 > 100 pg/mL) and are often contraindicated in subjects with significant kidney and liver injury [5]. Radiotherapy is commonly linked to malignant disease therapy. In such therapeutic option, high-dose RT is used and induces the production of proinflammatory cytokines, leading to an inflammatory response in the irradiated tissues. Contrary to this, RT administered at low doses (LDRT) modulates the inflammatory response. LDRT may act as an anti-inflammatory factor irrespective of the mechanism that had induced inflammation [6]. The mechanisms of the anti-inflammatory action of LDRT are complex. The main aspects include modulation of macrophage function and leukocyte recruitment, together with the induction of apoptosis in immune cells and a decrease in proinflamatory cytokine concentration [7,8]. This feature of LDRT has been known since the first half of the 20th century, prior to the antibiotics era, when this method was used against various inflammatory and infectious diseases. Clinical utility of LDRT in such cases involving pneumonia, sinusitis, arthritis, gas gangrene, inner ear infection or carbuncles has been well described by Calabrese [9]. The efficacy of LDRT has also been confirmed in the treatment of degenerative and inflammatory diseases of connective tissues, such as osteoarthritis, humeral epicondylitis, scapular–humeral periarthritis, or heel spurs [10,11]. Due to above facts, the concept of utilizing LDRT as a suppressor of COVID-19-related pneumonia has been raised. In this paper, the results of a two-phase study on the feasibility and safety of LDRT in patients with pneumonia in the course of COVID-19 together with preliminary results of the effectiveness of the method have been presented.

## 2. Material and Methods

This 2-phase study included 17 patients hospitalized between December 2020 and April 2021 due to severe viral pneumonia in the course of COVID-19 in Department of Infectious Diseases and Hepatology, Medical University of Silesia, Bytom, Poland. Out of this group, 15 patients fulfilled inclusion criteria and underwent the procedure. There were 6 females and 9 men in the median age of 66 years (range 49–78). All of them required continuous oxygen supplementation. Inclusion criteria consisted of COVID-19 infection confirmed by PCR in nasopharyngeal swab, age ≥ 18 years, Zubrod score ≤ 3 points, clinical and radiological (RTG or HRCT) signs of viral pneumonia, severe COVID-19 in stage 3 (pre-ARDS) according to national guidelines with SpO_2_ < 90% and the need for oxygen supplementation [12] and the ability to provide concise consent. Among exclusion criteria were ARDS, the need for invasive or mechanical ventilation, pregnancy, any thorax malignancy in the last 5 years, contraindication for medical transport for LDRT procedure, cognitive impairment and therapy with another experimental therapy. The study was conducted according to the guidelines of the Declaration of Helsinki and approved by the Ethics Committee of Maria Sklodowska-Curie National Research Institute of Oncology, Gliwice branch (decision code: KB/430-104/20 date of approval: 19 November 2020) and all participants gave written informed consent.

Radiotherapy procedure was performed in Radiotherapy Department of Maria Sklodowska-Curie National Research Institute of Oncology in Gliwice, Poland which is located about 20 km from the Department of Infectious Diseases where the patients had been hospitalized. All patients received a single-fraction radiation dose of 1 Gy to the bilateral lungs, which was delivered via an anterior–posterior and posterior–anterior beam configuration. The procedure of treatment preparation, dosimetric and quality control aspects of LDRT have been described elsewhere in detail [13]. Due to epidemiological requirements and poor general status of most of the patients, all treatment procedures were modified to be as short as possible not compromising quality assurance and were completed in 30 min, and the whole length of stay in Radiotherapy Department was not longer than 45 min with continuous oxygen support. After LDRT procedure, patient was transported back to Department of Infectious Diseases where they stayed until the end of hospitalization.

Despite routine medical care, according to protocol additional follow-up procedures were performed on day 1, 3, 5, 7, 14 after LDRT which included a physical examination and assessment of COVID-19 disease grade according to WHO scale and PTEILCHZ scoring system, O_2_ saturation, basic laboratory values, i.e., hematological (morphology), renal (creatine), hepatic (Aspat, Alat, bilirubin), coagulation (INR), and inflammatory biochemical markers, i.e., CRP (C-reactive protein, mg/dL), IL-6, ferritin, D-dimers, LDH. Chest RTG or HRCT was performed at baseline and between days 7 and 14. The decision about the treatment regimen was taken entirely by the treating physician concerning current knowledge and national recommendations [12]. If selected, remdesivir was administered intravenously once daily for 5 days, with a loading dose of 200 mg on day 1, followed by a maintenance dose of 100 mg; tocilizumab (in one patient) was administered iv in a single dose of 800 mg if PBW > 90 kg; 600 mg if PBW > 65 kg and ≤90 kg; 400 mg if PBW > 40 kg and ≤65 kg. Dexamethasone was usually administered orally or intravenously with doses 4–8 mg per day. All subjects received low molecular weight heparins in doses between 0.5 and 1.0 mg/kg.

### Statistical Analyses

Sample size has not been calculated. Data were presented as number and percentage or median and 25–75% Confidence Intervals (25–75% CI). The difference between time points was calculated by Wilcoxon signed-rank test and Friedman ANOVA tests. *p* value below 0.05 was considered statistically significant. Statistical analyses were performed by use of Statistica 13.0 (TIBCO Software Inc., Palo Alto, CA, USA) and GraphPad Prism 5.1 software (GraphPad Software, Inc., La Jolla, CA, USA).

## 3. Results

Seventeen consecutive COVID-19 patients fulfilling inclusion criteria gave written informed consent. Two patients who consented to the trial rapidly deteriorated, required intubation and could not undergo LDRT. Fifteen patients finally underwent the LDRT procedure. Patients shared multiple negative predictive factors including older age, median 66 years (range 49–78), obesity, median BMI 31 (CI: 28–35), comorbidities (13 patients had arterial hypertension, 8 diabetes, 3 kidney injury) and low baseline oxygen saturation (median 84%, CI: 81–86%). The average duration of symptoms prior to the LDRT procedure was 7 days (25–75% CI: 5–8 days). Comedications mainly consisted of low molecular weight heparin, dexamethasone and remdesivir. One subject was additionally treated with anti-SARS-Cov2 FFP and one with tocilizumab. Clinical characteristics of studied patients are included in Table 1.

### LDRT Efficacy

**Figure 1:**
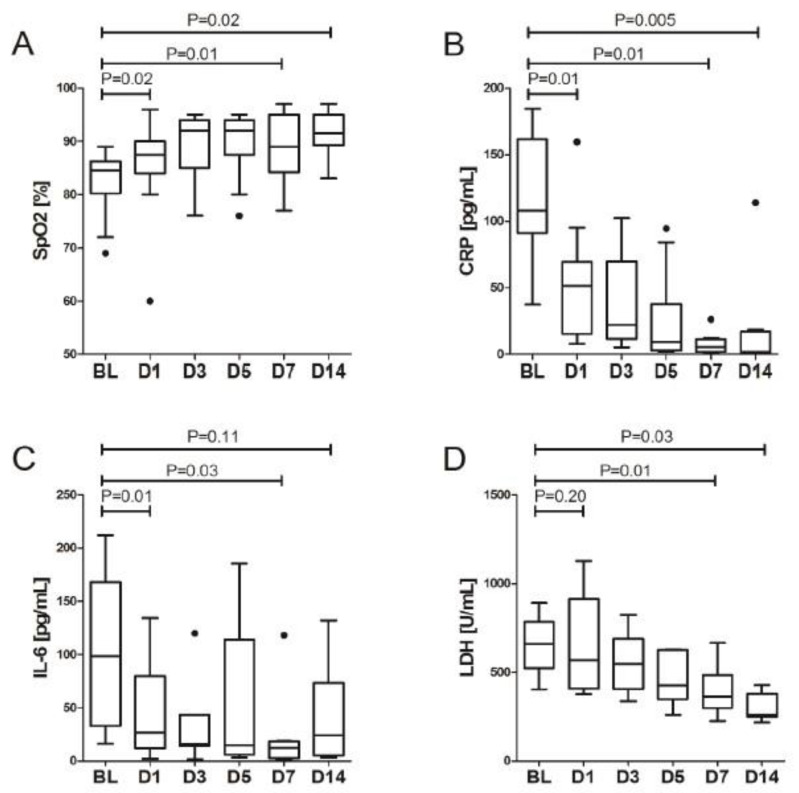
The change of concentration of selected parameters in the blood in subsequent days after LDRT. (**A**) O_2_ saturation, (**B**) C-reactive protein, (**C**) interleukin 6, (**D**) lactate dehydrogenase. Bold dots denote outliers.

In all subjects, LDRT was performed without early adverse events and procedure was well tolerated. Of 15 hospitalized patients, 11 (73%) were released from hospital prior to or on day 28. Median duration of hospitalization was 20 days (CI: 20–24). Three patients died due to progression of COVID-19, 28-day mortality was 13%. Importantly, median oxygen saturation (Sp0_2_) rapidly improved within the first 24 h post-LDRT in the majority of patients (14/15, 93%), with median Sp02 values of 84.5% (81.0–86.0) vs. 87.5% (84.0–90.0), *p* = 0.016, respectively. The trend was stable over the remaining days with a continuous increase for the duration of the study (ANOVA *p* = 0.007), Figure 1A. At day 14 of hospitalization, out of 13 living subjects, 6 (46%) did not require further oxygen supplementation (Table 1).

**Figure 2:**
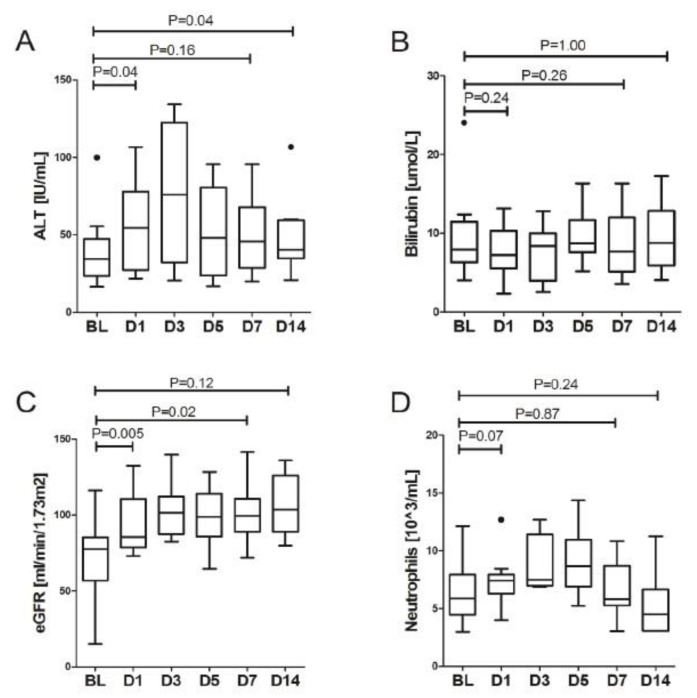
The change in concentration of selected parameters in the blood in subsequent days after LDRT: (**A**) alanin aminotransferaz, (**B**) bilirubin, (**C**) glomelural filtration, (**D**) neutrophils level. Bold dots denote outliers.

**Figure 3:**
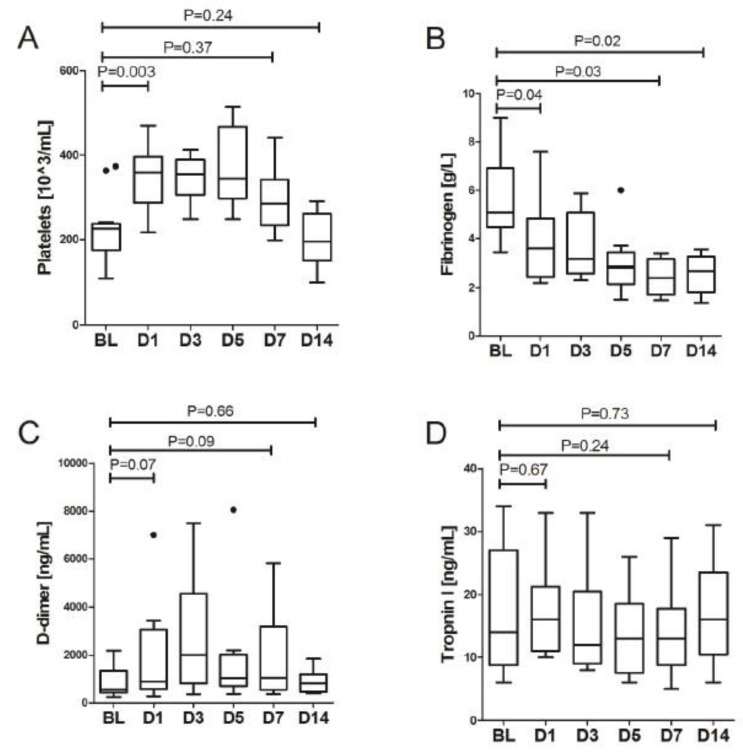
The change of concentration of selected parameters in the blood in subsequent days after LDRT: (**A**) platelets, (**B**) fibrinogen, (**C**) D-dimers, (**D**) troponin. Bold dots denote outliers.

Furthermore, we observed a significant improvement in the blood concentration of numerous proinflammatory parameters during 24 h after LDRT. These included a twofold decline in serum CRP (107.9 (91.2–161.5) vs. 51.4 (16.8–67.0), *p* = 0.007) and a threefold decline in serum IL-6 concentration (98.7 (32.8–168.3) vs. 26.9 (14.4–63.0), *p* = 0.006), Figure 1. Significant improvement during the first day was observed with WBC, fibrinogen and eGFR. Serum LDH showed slower dynamics of improvement with significant decrease observed only at day 3, Table 2. Overall, a decrease in biochemical response, expressed as statistically significant, was observed between baseline and day 7 values for CRP, IL-6, fibrinogen and LDH, and between day 0 and day 14 for CRP, fibrinogen, LDH and ferritin, Table 3. More importantly, there were no safety signals following the procedure, there was only a slight elevation of ALT with a peak on day 3, stable bilirubin concentration and neutrophil counts and improvement of eGFR thorough the study period, Figure 2. Additionally, no significant trends in the activation of coagulation nor cardiac involvement were observed with the exception of decrease of fibrinogen concentration in subsequent days after LDRT, Figure 3.

**Figure 4:**
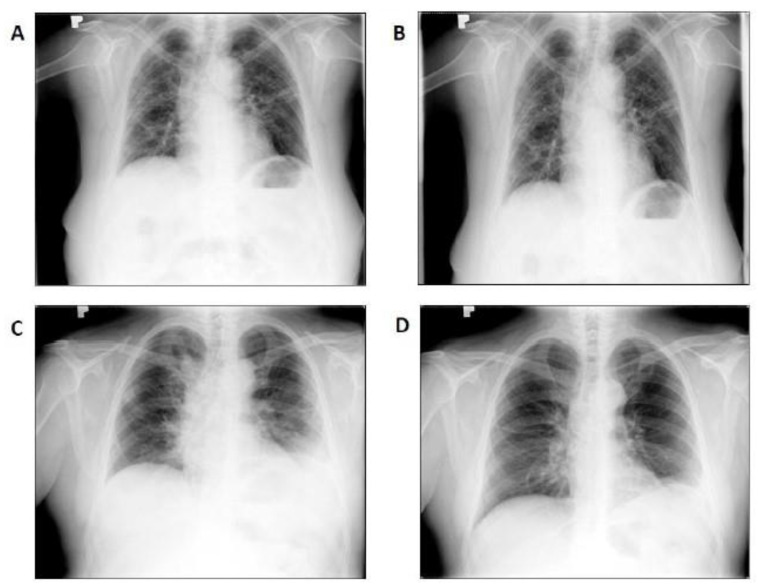
Chest radiographs of first two consecutive patients with COVID-19-associated pneumonia included in the study prior to and 14 days after LDRT procedure showing radiological improvement. ((**A**)—patient #1 baseline, (**B**)—patient #1 day 14, (**C**)—patient #2 baseline, (**D**)—patients #2 day 14).

Radiological improvement, expressed as the decrease in intensity of lung infiltrates, was observed in 10 (67%) of patients after 7–14 days, although exact comparison of area of infiltrates was not carried out due to the different modalities used (CXR and HRCT) as well as the timing frame. Examples of individual radiograms are shown in Figure 4.

## 4. Discussion

It has been ascertained that high concentrations of proinflammatory cytokines in the plasma known as a cytokine storm is responsible for the severity of infection and the high mortality in some patients with COVID-19 [14]. Most of these cytokines are liberated from macrophages due to COVID-19 interaction [15]. Although numerous medications have been suggested to cope with uncontrolled cytokine release and to reverse the course of the disease in patients, which lead to the need for mechanical ventilation, the mortality in the ARDS phase of COVID-19 usually exceeds 20% [16]. We have previously shown that in patients with severe COVID-19, particularly those developing cytokine storms, administration of tocilizumab significantly improved survival even when compared to dexamethasone [17]. Importantly, in another study, we found that the beneficial effects of tocliziumab were only present in patients with sP02 < 90% and a high baseline of serum IL-6 concentration > 100 pg/mL. In this group, the reduction in mortality was more than twofold after tocliziumab administration [18]. On the other hand, TCZ has not only a specific indication for use, but also important contraindications including levels of neutrophil, activity of ALT and kidney function. Low-dose radiation therapy seems to be more universally applicable in the settings of COVID-19.

It has been well documented that LDRT suppresses inflammation through various mechanisms, including the modulatory effect on endothelial cells, induction of apoptosis in immune cells, the secretion of anti-inflammatory factors or shifting macrophage function and increasing natural killer cell activity and interferon production [7,8,19,20]. There are over 6600 clinical trials on COVID-19, but only 18 of them consider the utility of LDRT against COVID-19 pneumonia. Out of these trials, 7 have been signed as completed and 11 are still recruiting (clinicaltrials.gov). Dunlap et al. carried out a PubMed search with key words “low dose radiation therapy” and “COVID-19” on 16 August 2021. Sixty-six publications were found to be related to the potential therapy for patients with COVID-19. Various potential mechanisms and rationales of LDRT action impacting COVID-19 patients has been proposed in most of the analyzed publications. Almost one-third of these publications concentrated on the potential application of LDRT for COVID-19 patients, while only 15% were clinical observations or trials [20]. Our results indicate that LDRT is a safe procedure and gives fast and significant improvements in oxygen saturation within 24 h after LDRT. Moreover, there was also immediate decrease in inflammatory biomarkers in 24 h and subsequently on days 7 and 14. Similar effects have been reported by other authors.

The presented results confirm the preliminary information about the utility of LDRT in COVID-19 pneumonia published by Hess et al., who also noticed immediate improvements in oxygen saturation in 24 h after a single fraction of 1.5 Gy of X-rays applied to both lungs in 3/5 (60%) of their patients. The same clinicians in the following two-phase study compared results of 10 patients who underwent LDRT with 10 control patients blindly matched by age and comorbidity. Patients after LDRT recovered to room air in 3 days, which was significantly shorter than the 12 days reported for the control cohort. In accordance with our findings, significantly reduced levels of the inflammatory biomarkers such as CRP and LDH also were found in their group after LDRT, which may indicate its modulatory effect on immune cells [21].

Ameri et al. reported the results of five patients who underwent LDRT with a single fraction of 0.5 Gy. Four patients (80%) showed initial improvement in O_2_ saturation and body temperature within 1 day after irradiation. The mean time to discharge was 6 days for three patients. One patient died on the third day after irradiation. No acute toxicity was found [22].

Sanmamed reported results of LDRT of 1 Gy in a single fraction in nine patients. Median time to receive RT from the date of admission was 52 days and most of the patients had anti-COVID treatment before. SatO_2_/FiO_2_ index significantly improved in 72 hours and 1 week after LDRT (*p* = 0.01). Out of other inflammatory biomarkers, only LDH significantly decreased 1 week after LDRT (*p* = 0.04). Two patients died, one due to sepsis and the other due to severe baseline chronic obstructive pulmonary disease from COVID-19 pneumonia [23].

Sharma et al. reported results of LDRT with a dose of 0.7 Gy in a single fraction in 10 patients. Nine of them had complete clinical recovery, mostly within a period ranging from 3 to 7 days. One patient showed clinical deterioration and died 24 days after LDRT. No acute radiation toxicity was observed [24].

In another trial from India, Ganesan et al. reported 25 patients with COVID-19-related pneumonia who underwent single-fraction LDRT of 0.5 Gy. There was a statistically significant improvement in oxygenation between pre-LDRT and day 2, 3 and day 7 after LDRT. Demand for supplemental oxygen was significantly reduced between pre-LDRT and day 2, 3 and day 7 after LDRT. Eighty-eight percent of patients attained clinical recovery within 10 days after LDRT. No acute toxicity was found in this study either [25].

Bonet et al. treated 36 patients with 0.5 Gy LDRT along with dexamethasone. They were able to complete the LDRT procedure from the first to the last step in a median time of 38 min. Shortening the time of the procedure seems to be very important due to the poor general condition of these patients. Significant improvements in oxygen saturations and the amount of supplemental oxygen needed were reached in these patients. CT scan taken at 1 week after LDRT revealed significant improvements in the percentage of lung involvement in those who survived [26].

All the above studies confirm the safety and effectiveness of LDRT due to pneumonia in patients with COVID-19. Contrary to these encouraging results, Papachristofilou et al. failed to improve clinical outcomes in 22 patients randomized to either whole-lung 1 Gy LDRT or sham-RT in the group of patients requiring mechanical ventilation as an inclusion criterium. Treatment of dexamethasone in both arms together with the fact that patients were generally elderly and comorbid, with a median age of 75 years, may partly explain the lack of benefit of LDRT in this group. However, the main reason for the poor results in this group was probably the fact that LDRT prevented rather than reduced the cytokine storm. Cytokine storms consequently turn into ARDS, in which the patient requires mechanical ventilation [27].

Some ideas to combine LDRT with other treatment options have also been proposed. Convalescent plasma contains neutralizing antibodies against COVID-19. LDRT can induce anti-inflammatory responses. The possible synergistic interactions of both may give clinical benefit for patients with COVID-19. The strategy of such combined therapy has been proposed by Abdollahi et al. [28]. 2-deoxy-D-glucose (2-DG) seems to act as a polypharmacological agent for COVID-19 treatment due to its effects on the glycolytic pathway, its presented anti-inflammatory action and its interaction with viral proteins. It has been suggested that 2-DG may increase the efficacy of LDRT in the treatment of COVID-19 pneumonia by adjuvant enhancement [29].

There is also some evidence, from preclinical and clinical data, suggesting that beside the anti-inflammatory effects, bacterial pneumonia could also be controlled by LDRT. If it is true, reducing bacterial co-infections could be an additional benefit for patients with COVID-19 [30]. 

It also has been hypothesized that LDRT would reduce or prevent blood clotting through reducing oxidative stress, decreasing the risk of microclots in the lungs of COVID-19-infected patients which, when larger in size, can migrate to the brain or heart, causing a stroke or heart attack [31].

Despite a few reports supporting the idea of utilizing LDRT for patients with pneumonia due to COVID-19, the body of evidence for a proposed beneficial effect of low-dose radiation on viral pneumonia is limited according to others. Many believe that radiation risk has no threshold and is linear with dosage. Few studies found a causal association between both low- and high-dose radiation exposure and most types of circulatory disease [32]. In diagnostic imaging, although the benefits outweigh the potential risks associated with low-dose radiation, the cumulative absolute risks are low. The delivered cumulative doses of about 50 mGy might almost triple the risk of leukemia and doses of about 60 mGy might triple the risk of brain cancer [33]. In recent years, however, growing evidence indicates the weaknesses of the LNT hypothesis. Authors indicated that the LNT model and its use for assessing the risks associated with low doses are not based on scientific evidence and even beneficial effects of hormesis for LDRT could be suspected [34,35,36,37]. Moreover, low-dose radiation exposure appeared to be not adequate for estimating the risk of cancer induction from radiotherapy for malignant or nonmalignant diseases [38].

Ghadimi-Moghadam points out another important feature of LDRT in COVID-19 patients. Due to SARS-COV-2’s high mutation rate, any antiviral drug against SARS-CoV-2 would exert an intense selective pressure on the virus which may turn into highly adaptive and treatment-resistant virus types with enhanced pathogenicity. According to Ghadimi-Moghadam, a single dose of 100, 180 or 250 mGy X-rays, which is less than the maximum annual radiation dose of the residents of the high-background-radiation area of Ramsar (<260 mSv), are safe and may prevent selective pressure and hence do not lead to a direct, accelerated evolution of these viruses [39].

Host cell damage is one of the results of COVID-19 infection leading to organ failure and serious life-threatening complications. Mesenchymal stromal cell (MSC) therapies are emerging as promising therapeutic interventions in patients with ARDS and sepsis due to their reparative, immunomodulatory and antimicrobial properties [40]. The capacity of LDRT to stimulate bone marrow progenitor cells, its proliferation and peripheral blood mobilization, and its therapeutic effects on damaged tissues have also been described [41]. As described by Roger et al., multiple in vivo studies in animal models and ex vivo human lung models have demonstrated the MSCs’ impressive capacity to inhibit lung damage, reduce inflammation and aid with alveolar fluid clearance. They also act in anti-lung fibrosis, anti-apoptosis of injured cells and lung tissue regeneration. Additionally, MSCs produce molecules that are antimicrobial and reduce pain. When administered intravenously, they travel directly to the lungs. Human clinical trials also included studies of acute respiratory distress syndrome. Recently, the application of MSCs in patients with pulmonary complications due to COVID-19 has demonstrated reduced patient mortality and, in some cases, improved long-term survival. These studies suggest that LDRT could be a possible mediator to improve stem cells in potential therapy for patients with COVID-19 [42].

Our study has several weaknesses: non-randomized design, experimental intent, small patient numbers, additional treatment in few patients and limited imaging. All these limitations make the exact magnitude of the benefit of LDRT uncertain, and this should be explored in randomized trials. Despite this, we are convinced that for the target population of predominantly older patients with pre-existing comorbidities LDRT is a safe and effective option. Moreover, LDRT is available in many centers without the need for a high financial investment and may reduce the overload of the health system, especially Intensive Care Units.

Our study confirms that a single-fraction radiation dose of 1 Gy to the bilateral lungs is feasible and well tolerated. LDRT may translate into early clinical recovery and is a promising treatment option in carefully selected patients with COVID-19 pneumonia. Furthermore, LDRT can be used in patients with kidney and liver damage, which makes this technique commonly applicable. These findings need to be validated by prospective randomized trials to better define candidates for LDRT and to explore the distant effects of this approach.

## Figures and Tables

**Table 1:** Baseline characteristics and day 28 clinical outcome of patients treated with low-dose radiation therapy for severe COIVD-19.

No	Age [yrs]	Gender (M/F)	BMI	Co-Morbidities	Symptoms Duration before LDRT	Co-Medications for COVID-19	Baseline MEWS [Points]	The Lowest Sp02 [%], Days after LDRT. *—Oxygen Support					Duration of Hospitalization [Days]	Outcome, Day 28	Outcome, Long-term Follow-Up
								BL	1	3	7	14			
1	49	M	n/a	AH, DM	10 days	RDV, LMWH, DEX	1	88 *	90 *	94 *	97 *	92	20	Released	Resolved
2	62	F	26	AH	6 days	DEX, LMWH, RDV, FFP	1	86 *	89 *	92 *	95 *	97	24	Released	Resolved
3	67	M	28	AH, DM	3 days	RDV, LMWH, DEX	1	69 *	87 *	85 *	84 *	90 *	28	Released	Resolved
4	70	F	n/a	AH, dyslipidemia, stroke	7 days	FFP, LMWH, DEX, RDV	1	82 *	84 *	76 *	85 *	89 *	28	Released	Resolved
5	78	M	n/a	AH emphysema	8 days	RDV, LMWH, DEX	3	85 *	86 *				11	Died	Died
6	68	M	31	AH	8 days	RDV, LMWH, DEX	1	78 *	84 *				6	Died	Died
7	69	F	39	AH, DM	9 days	LMWH	1	85 *	96 *	95 *	95 *	95	24	Released	Resolved
8	61	M	31	Acute kidney injury	5 days	LMWH, DEX	3	83 *	88 *	83 *	83 *		14	Mechanical ventilation	Died
9	66	F	26	AH, DM atrial fibrillation	8 days	DEX, LMWH	1	86 *	90 *	94 *	86		15	Released	Resolved
10	68	M	34	AH, dyslipidemia,	7 days	LMWH, DEX	1	84 *	60 *	85 *	85 *		20	Released	Resolved
11	65	M	35	Hypothyroidism, CKD	7 days	RDV, LMWH, DEX	1	72 *	80 *	80 *			7	High flow	Lost to follow-up
12	53	F	30	AH, DM	5 days	RDV, LMWH, DEX	1	76 *	80 *	82 *	77 *	83 *	23	Released	Resolved
13	56	F	31	AH, DM, CKD, cardiomyopathy, pulmonary embolism	5 days	RDV, LMWH, DEX	1	81 *	86 *	95 *	95		14	Released	Resolved
14	53	M	35	AH, DM, liver damage	14 days	LMWH, DEX	1	89 *	96 *	82 *	95 *	95	10	Released	Resolved
15	74	M	31	AH, DM, UTI E. faecium VRE+	1 day	RDV, TCZ, DEX, LMWH	1	87 *	88 *	92 *	92 *	91 *	28	Released	Resolved

Abbreviations: AH—arterial hypertension, DM—diabetes mellitus, CKD—chronic kidney disease, UTI—urinary tract infection, RDV—remdesivir, DEX—dexamethasone, LMWH—low molecular weight heparin, FFP—anti-SARS-Cov-2 fresh frozen plasma, TCZ—tocilizumab.

**Table 2:** Dynamics of serum proinflammatory markers and safety parameters in subjects treated with low-dose radiation therapy for severe COVID-19 (median, 25–75% CI, P statistical significance vs. previous timepoint).

	Baseline	Day 1	*p*	Day 3	*p*	Day 5	*p*	Day 7	*p*	Day 14	*p*	Friedman ANOVA P
Sp02 [%]	84.5 (81.0–86.0)	87.5 (84.0–90.0)	0.016	92.0 (85.0–94.0)	0.308	92.0 (90.0–94.0)	0.463	89.0 (84.5–95.0)	0.575	91.5 (89.5–95.0)	0.249	0.007
WBC [10^3^/uL]	7.3 (5.4–9.2)	8.7 (8.1–9.4)	0.028	8.4 (8.3–9.6)	0.345	10.1 (8.1–13.0)	0.463	7.8 (7.3–11.2)	0.575	7.6 (5.8–9.9)	0.036	0.702
Neutrophils [10^3^/uL]	5.9 (4.6–7.9)	7.4 (6.5–7.5)	0.069	7.5 (7.1–10.2)	0.180	8.6 (7.0–10.9)	n/a	5.8 (5.3–8.7)	0.285	4.5 (3.1–6.6)	0.080	n/a
Platelets [10^3^/uL]	225 (175–237)	359 (290–288)	0.003	354 (310–387)	0.753	344 (297–467)	0.465	285 (251–339)	0.025	196 (163–257)	0.017	0.214
CRP [ng/mL]	107.9 (91.2–161.5)	51.4 (16.8–67.0)	0.007	22.1 (13.3–68.2)	0.017	9.3 (3.5–33.4)	0.110	5.5 (2.2–10.7)	0.018	1.9 (0.6–16.7)	0.237	<0.001
IL-6 [pg/mL]	98.7 (32.8–168.3)	26.9 (14.4–63.0)	0.006	15.8 (14.1–43.5)	0.138	14.7 (5.8–113.0)	0.500	12.2 (3.4–18.5)	0.674	24.2 (5.6–53.9)	0.080	0.119
Fibrinogen [g/L]	5.1 (4.6–5.8)	3.6 (2.5–4.7)	0.037	3.2 (2.7–4.7)	0.018	2.8 (2.2–3.4)	0.075	2.4 (1.8–3.0)	0.028	2.7 (1.9–3.1)	0.600	0.233
D-dimer [ng/mL]	561 (434–1351)	890 (577–3054)	0.075	2012 (819–3628)	0.401	1028 (700–2027)	0.093	1044 (596–2325)	0.161	809 (470–1195)	0.021	0.625
LDH [U/L]	660 (523–785)	570 (419–875)	0.203	547 (421-679)	0.028	425 (349–626)	0.028	364 (307–480)	0.008	262 (247–378)	0.028	0.017
Ferritin [ng/mL]	2453 (912–2717)	1128 (686–2456)	0.249	1607 (390–2125)	0.080	1106 (534–2251)	1.000	797 (453–1992)	0.267	720 (370–992)	0.068	n/a
ALT [IU/mL]	34.3 (25.7–45.3)	54.4 (27.6–76.2)	0.041	75.9 (32.8–114.9)	0.327	48.0 (25.0–76.9)	0.889	45.7 (28.8–66.0)	1.000	40.4 (36.1–59.0)	0.674	0.924
Bilirubin [umol/L]	7.9 (6.5–10.6)	7.2 (5.6–10.1)	0.241	8.4 (4.4–9.9)	0.600	8.7 (7.6–11.6)	0.753	7.6 (5.3–11.4)	0.735	8.8 (6.1–12.5)	0.779	0.490
Troponin T [ng/L]	14 (9–26)	16 (11–19)	0.675	12 (9–16)	0.345	13 (9–14)	0.273	13 (9–16)	0.059	16 (11–22)	0.012	0.428
eGFR [ml/min/1.73 m^2^]	77.4 (63.8–84.8)	85.6 (79.1–107.9)	0.005	101.6 (89.0–110.8)	0.018	98.7 (86.9–113.1)	0.249	99.4 (89.1–110.5)	0.173	103.4 (89.0–125.5)	0.674	0.119

**Table 3:** Dynamics of serum proinflammatory markers and safety parameters in subjects treated with low-dose radiation therapy for severe COVID-19 (median, IQR, P statistical significance baseline vs. day 7 and day 14).

	Baseline	Day 7	*p*	Day 14	*p*
Sp02 [%]	84.5 (81.0–86.0)	89.0 (84.5–95.0)	0.008	91.5 (89.5–95.0)	0.018
WBC [10^3^/uL]	7.3 (5.4–9.2)	7.8 (7.3–11.2)	0.214	7.61 (5.79–9.93)	0.799
Neutrophils [10^3^/uL]	5.9 (4.6–7.9)	5.8 (5.3–8.7)	0.866	4.49 (3.07–6.63)	0.237
Platelets [10^3^/uL]	225 (175–237)	285 (251–339)	0.374	195.5 (163.0–257.0)	0.241
CRP [ng/mL]	107.9 (91.2–161.5)	5.5 (2.2–10.7)	0.008	1.91 (0.60–16.70)	0.005
IL-6 [pg/mL]	98.7 (32.8–168.3)	12.2 (3.4–18.5)	0.028	24.2 (5.6–53.9)	0.116
Fibrinogen [g/L]	5.1 (4.6–5.8)	2.4 (1.8–3.0)	0.028	2.7 (1.9–3.1)	0.018
D-dimer [ng/mL]	561 (434–1351)	1044 (596–2325)	0.093	809.0 (469.7–1194.9)	0.657
LDH [U/L]	660 (523–785)	364 (307–480)	0.008	262 (247–378)	0.028
Ferritin [ng/mL]	2453 (912–2717)	797 (453–1992)	0.173	720.2 (369.7–992.1)	0.043
ALT [IU/mL]	34.3 (25.7–45.3)	45.7 (28.8–66.0)	0.161	40.4 (36.1–59.0)	0.037
Bilirubin [umol/L]	7.9 (6.5–10.6)	7.6 (5.3–11.4)	0.263	8.8 (6.1–12.5)	1.000
Tropnin T [ng/L]	14 (9–26)	13 (9–16)	0.249	16.0 (11.0–22.0)	0.753
eGFR [mL/min/1.73 m^2^]	77.4 (63.8–84.8)	99.4 (89.1–110.5)	0.005	103.4 (89.0–125.5)	0.005

## Data Availability

Available in contact with corresponding author.

## References

[1] Research Result. https://www.clinicaltrials.gov/ct2/home.

[2] Ruan Q., Yang K., Wang W., Jiang L., Song J. (2020). Clinical predictors of mortality due to COVID-19 based on an analysis of data of 150 patients from Wuhan, China. Intensive Care Med..

[3] Yang X., Yu Y., Xu J., Shu H., Liu H., Wu Y., Zhang L., Yu Z., Fang M., Yu T. (2020). Clinical course and outcomes of critically ill patients with SARS-CoV-2 pneumonia in Wuhan, China: A single-centered, retrospective, observational study. Lancet Respir. Med..

[4] Ñamendys-Silva S. (2020). Respiratory support for patients with COVID-19 infection. Lancet Respir. Med..

[5] Salama C., Han J., Yau L., Reiss W.G., Kramer B., Neidhart J.D., Criner G.J., Kaplan-Lewis E., Baden R., Pandit L. (2021). Tocilizumab in Patients Hospitalized with Covid-19 Pneumonia. N. Engl. J. Med..

[6] Rödel F., Keilholz L., Herrmann M., Sauer R., Hildebrandt G. (2007). Radiobiological mechanisms in inflammatory diseases of low-dose radiation therapy. Int. J. Radiat. Biol..

[7] Rodel F., Frey B., Gaipl U., Keilholz L., Fournier C., Manda K., Schollnberger H., Hildebrandt G., Rodel C. (2012). Modulation of inflammatory immune reactions by low-dose ionizing radiation: Molecular mechanisms and clinical application. Curr. Med. Chem..

[8] Arenas M., Sabater S., Hernández V., Rovirosa A., Lara P.C., Biete A., Panés J. (2012). Anti-inflammatory effects of low-dose radiotherapy. Indications, dose, and radiobiological mechanisms involved. Strahlenther. Onkol..

[9] Calabrese E.J., Dhawanet G. (2013). How radiotherapy was historically used to treat pneumonia: Could it be useful today?. J. Biol. Med..

[10] Micke O., Seegenschmied M.H. (2002). German Working Group on Radiotherapy in Germany. Consensus guidelines for radiation therapy of benign diseases: A multicenter approach in Germany. Int. J. Radiat. Oncol. Biol. Phys..

[11] Seegenschmiedt M.H., Katalinic A., Makoski H., Haase W., Gademann G., Hassenstein E. (2000). Radiation therapy for benign diseases: Patterns of care study in Germany. Int. J. Radiat. Oncol. Biol. Phys..

[12] Flisiak R., Horban A., Jaroszewicz J., Kozielewicz D., Pawłowska M., Parczewski M., Piekarska A., Simon K., Tomasiewicz K., Zarębska-Michaluk D. (2020). Management of SARS-CoV-2 infection: Recommendations of the Polish Association of Epidemiologists and Infectiologists as of March 31, 2020. Pol. Arch. Intern. Med..

[13] Ślosarek K., Gądek A., Sroka Ł., Dolla Ł., Biały A., Radwan M., Bodusz D., Nachlik M., Rutkowski T., Jaroszewicz J. (2021). Lung volume irradiation procedures in patients with pneumonia during COVID-19 infection—Physical aspects of treatment planning and dosimetry. Nowotwory J. Oncol..

[14] Huang C., Wang Y., Li X. (2020). Clinical features of patients infected with 2019 novel coronavirus in Wuhan, China. Lancet.

[15] Wong C.K., Lam C.W.K., Wu A.K.L., Ip W.K., Lee N.L.S., Chan I.H.S., Lit L.C.W., Hui D.S.C., Chan M.H.M., Chung S.S.C. (2004). Plasma inflammatory cytokines and chemokines in severe acute respiratory syndrome. Clin. Exp. Immunol..

[16] Flisiak R., Rzymski P., Zarębska-Michaluk D., Rogalska M., Rorat M., Czupryna P., Lorenc B., Ciechanowski P., Kozielewicz D., Piekarska A. (2021). Demographic and Clinical Overview of Hospitalized COVID-19 Patients during the First 17 Months of the Pandemic in Poland. J. Clin. Med..

[17] Zarębska-Michaluk D., Jaroszewicz J., Rogalska M., Martonik D., Pabjan P., Berkan-Kawińska A., Bolewska B., Oczko-Grzesik B., Kozielewicz D., Tudrujek-Zdunek M. (2021). Effectiveness of Tocilizumab with and without Dexamethasone in Patients with Severe COVID-19: A Retrospective Study. J. Inflamm. Res..

[18] Flisiak R., Jaroszewicz J., Rogalska M., Łapiński T., Berkan-Kawińska A., Bolewska B., Tudrujek-Zdunek M., Kozielewicz D., Rorat M., Leszczyński P. (2021). Tocilizumab Improves the Prognosis of COVID-19 in Patients with High IL-6. J. Clin. Med..

[19] Schröder S., Kriesen S., Paape D., Hildebrandt G., Manda K. (2018). Modulation of Inflammatory Reactions by Low-Dose Ionizing Radiation: Cytokine Release of Murine Endothelial Cells Is Dependent on Culture Conditions. J. Immunol. Res..

[20] Dunlap N.E., van Berkel V., Cai L. (2021). COVID-19 and low-dose radiation therapy. Radiat. Med. Prot..

[21] Hess C.B., Nasti T.H., Dhere V.R., Kleber T.J., Switchenko J.M., Buchwald Z.S., Stokes W.A., Weinberg B.D., Rouphael N., Steinberg J.P. (2021). Immunomodulatory Low-Dose Whole-Lung Radiation for Patients with Coronavirus Disease 2019-Related Pneumonia. Int. J. Radiat. Oncol. Biol. Phys..

[22] Ameri A., Ameri P., Rahnama N., Mokhtari M., Sedaghat M., Hadavand F., Bozorgmehr R., Haghighi M., Taghizadeh-Hesary F. (2021). Low-Dose Whole-Lung Irradiation for COVID-19 Pneumonia: Final Results of a Pilot Study. Int. J. Radiat. Oncol. Biol. Phys..

[23] Sanmamed N., Alcantara P., Cerezo E., Gaztañaga M., Cabello N., Gómez S., Bustos A., Doval A., Corona J., Rodriguez G. (2021). Low-Dose Radiation Therapy in the Management of Coronavirus Disease 2019 (COVID-19) Pneumonia (LOWRAD-Cov19): Preliminary Report. Int. J. Radiat. Oncol. Biol. Phys..

[24] Sharma D.N., Guleria R., Wig N., Mohan A., Rath G., Subramani V., Bhatnagar S., Mallick S., Sharma A., Patil P. (2021). Low-dose radiation therapy for COVID-19 pneumonia: A pilot study. Br. J. Radiol..

[25] Ganesan G., Ponniah S., Sundaram V., Marimuthu P.K., Pitchaikannu V., Chandrasekaran M., Thangarasu J., Kannupaiyan G., Ramamoorthy P., Thangaraj B. (2021). Whole lung irradiation as a novel treatment for COVID-19: Interim results of an ongoing phase 2 trial in India. Radiother. Oncol..

[26] Bonet M., Vázquez S., García E., Visus M., Jové D., Ripol O., Solé C., Gutiérrez L., Morales-Rull J.L., Montero Á. (2021). Saving time in the radiotherapy procedures for COVID-19 pneumonia treatment. A single-institution experience. Clin. Transl. Oncol..

[27] Papachristofilou A., Finazzi T., Blum A., Zehnder T., Zellweger N., Lustenberger J., Bauer T., Dott C., Avcu Y., Kohler G. (2021). Low-Dose Radiation Therapy for Severe COVID-19 Pneumonia: A Randomized Double-Blind Study. Int. J. Radiat. Oncol. Biol. Phys..

[28] Abdollahi H., Shiri I., Bevelacqua J.J., Jafarzadeh A., Rahmim A., Zaidi H., Mortazavi S.A.R., Mortazavi S.M.J. (2020). Low Dose Radiation Therapy and Convalescent Plasma: How a Hybrid Method May Maximize Benefits for COVID-19 Patients. J. Biomed. Phys. Eng..

[29] Verma A., Adhikary A., Woloschak G., Dwarakanath B.S., Papineni R.V. (2020). A combinatorial approach of a polypharmacological adjuvant 2-deoxy-D-glucose with low dose radiation therapy to quell the cytokine storm in COVID-19 management. Int. J. Radiat. Biol..

[30] Yang G., Kong Q., Wang G., Jin H., Zhou L., Yu D., Niu C., Han W., Li W., Cui J. (2014). Low-dose ionizing radiation induces direct activation of natural killer cells and provides a novel approach for adoptive cellular immunotherapy. Cancer Biother. Radiopharm..

[31] Bevelacqua J.J., Mortazavi S.A.R., Mortazavi S.M.J. (2020). Re: Low dose radiation therapy for COVID-19 pneumonia: Is there any supportive evidence?. Int. J. Radiat. Biol..

[32] Little M.P. (2016). Radiation and circulatory disease. Mutat. Res. Rev..

[33] Pearce M.S., Salotti J.A., Little M.P., McHugh K., Lee C., Kim K.P., Howe N.L., Ronckers C.M., Rajaraman P., Craft A.W. (2012). Radiation exposure from CT scans in childhood and subsequent risk of leukaemia and brain tumours: A retrospective cohort study. Lancet.

[34] Schirrmacher V. (2021). Less Can Be More: The Hormesis Theory of Stress Adaptation in the Global Biosphere and Its Implications. Biomedicines.

[35] Cuttler J.M. (2014). Remedy for radiation fear—Discard the politicized science. Dose Respons..

[36] Jaworowski Z. (2010). Radiation hormesis—A remedy for fear. Hum. Exp. Toxicol..

[37] Tubiana M., Feinendegen L.E., Yang C., Kaminski J.M. (2009). The linear no-threshold relationship is inconsistent with radiation biologic and experimental data. Radiology.

[38] Trott K.R., Kamprad F. (2006). Estimation of cancer risks from radiotherapy of benign diseases. Strahlenther. Onkol..

[39] Ghadimi-Moghadam A., Haghani M., Bevelacqua J.J., Jafarzadeh A., Kaveh-Ahangar A., Mortazavi S.M.J., Mortazavi S.A.R. (2020). SAR COVID-19 Tragic Pandemic: Concerns over Unintentional “Directed Accelerated Evolution” of Novel Coronavirus (SARS-CoV-2) and Introducing a Modified Treatment Method for ARDS. J. Biomed. Phys. Eng..

[40] Gorman E., Millar J., McAuley D., O’Kane C. (2021). Mesenchymal stromal cells for acute respiratory distress syndrome (ARDS), sepsis, and COVID-19 infection: Optimizing the therapeutic potential. Expert Rev. Respir. Med..

[41] Wang G.J., Cai L. (2000). Induction of cell-proliferation hormesis and cell-survival adaptive response in mouse hematopoietic cells by whole-body low-dose radiation. Toxicol. Sci..

[42] Rogers C.J., Harman R.J., Bunnell B.A., Schreiber M.A., Xiang C., Wang F.S., Santidrian A.F., Minev B.R. (2020). Rationale for the clinical use of adipose-derived mesenchymal stem cells for COVID-19 patients. J. Transl. Med..

